# Connectivity of high-frequency bursts as SOZ localization biomarker

**DOI:** 10.3389/fnetp.2024.1441998

**Published:** 2024-09-20

**Authors:** Marco Pinto-Orellana, Beth Lopour

**Affiliations:** Biomedical Engineering Department, University of California, Irvine, Irvine, CA, United States

**Keywords:** seizure onset zone, intracranial EEG, connectivity, gabor transform, robust filtering, epilepsy, high frequency oscillation, ripple

## Abstract

For patients with refractory epilepsy, the seizure onset zone (SOZ) plays an essential role in determining the specific regions of the brain that will be surgically resected. High-frequency oscillations (HFOs) and connectivity-based approaches have been identified among the potential biomarkers to localize the SOZ. However, there is no consensus on how connectivity between HFO events should be estimated, nor on its subject-specific short-term reliability. Therefore, we propose the channel-level connectivity dispersion (CLCD) as a metric to quantify the variability in synchronization between individual electrodes and to identify clusters of electrodes with abnormal synchronization, which we hypothesize to be associated with the SOZ. In addition, we developed a specialized filtering method that reduces oscillatory components caused by filtering broadband artifacts, such as sharp transients, spikes, or direct current shifts. Our connectivity estimates are therefore robust to the presence of these waveforms. To calculate our metric, we start by creating binary signals indicating the presence of high-frequency bursts in each channel, from which we calculate the pairwise connectivity between channels. Then, the CLCD is calculated by combining the connectivity matrices and measuring the variability in each electrode’s combined connectivity values. We test our method using two independent open-access datasets comprising intracranial electroencephalography signals from 89 to 15 patients with refractory epilepsy, respectively. Recordings in these datasets were sampled at approximately 1000 Hz, and our proposed CLCDs were estimated in the ripple band (80–200 Hz). Across all patients in the first dataset, the average ROC-AUC was 0.73, and the average Cohen’s d was 1.05, while in the second dataset, the average ROC-AUC was 0.78 and Cohen’s d was 1.07. On average, SOZ channels had lower CLCD values than non-SOZ channels. Furthermore, based on the second dataset, which includes surgical outcomes (Engel I-IV), our analysis suggested that higher CLCD interquartile (as a measure of CLCD distribution spread) is associated with favorable outcomes (Engel I). This suggests that CLCD could significantly assist in identifying SOZ clusters and, therefore, provide an additional tool in surgical planning for epilepsy patients.

## 1 Introduction

Epilepsy is a neurological disorder characterized by a tendency to have spontaneous seizures Milligan (2021); Dredge (2022). With 3 million adults and 470.000 children affected by epilepsy in 2015 in the United States, this condition is the fourth most frequent neurological disorder (Zack and Kobau, 2017). Despite its prevalence, only 36.36% of the patients diagnosed with epilepsy report successful management of their condition via the use of medications or surgery, and this ratio seems to be unaffected by factors such as age or sex (Kobau et al., 2023).

When multiple anti-seizure medications fail to control seizures, patients are deemed to have drug-resistant epilepsy (DRE), and they may consider surgery in the hopes of achieving seizure freedom. Current surgical treatments are based on the concept of an epileptogenic zone (EZ), defined as “the area of cortex that is necessary and sufficient for initiating seizures and whose removal (or disconnection) is necessary for complete abolition of seizures” (Jehi, 2018; Lüders et al., 2006; Rosenow and Lüders, 2001). There are currently no biomarkers to delineate the EZ before the surgery; therefore, the brain region in which seizure activity first occurs, known as the seizure onset zone (SOZ), is often used as the best evidence for determining which regions to remove. However, removing the identified SOZ alone is often not enough to achieve complete independence from seizures in DRE patients (Jehi, 2018). Considering that localizing the SOZ is challenging, even for experienced clinicians (Li et al., 2021), there is a need to develop new biomarkers to improve SOZ localization and provide additional evidence of regions within the EZ. This study focuses on the former task by providing a new biomarker based on intracranial EEG (iEEG) connectivity to assist in SOZ identification.

High-frequency oscillations (HFOs) are often defined as distinguishable spontaneous bursts with frequencies ranging between 80 Hz and 500 Hz, consisting of at least four cycles (Zijlmans et al., 2017). While these oscillations are observed in normal physiological processes (Lachaux et al., 2012), changes in HFO properties can be associated with seizure-generating tissue (often termed “pathological HFOs”) (Cimbalnik et al., 2018), and seizure dynamics (Schönberger et al., 2019). Although several cellular and network mechanisms are hypothesized to generate pathological HFOs (Jiruska et al., 2017), the precise role of HFOs in seizures remains an active area of research.

HFO rates have shown promise in identifying the SOZ (Thomschewski et al., 2019; Charupanit et al., 2020). However, their potential as SOZ biomarkers and the relationship between HFOs and the SOZ is still under debate (Lee et al., 2020), as some studies reported inconsistent findings (Jacobs et al., 2018). It is important to note that technical factors, such as frequency filter design and identification algorithms, may influence HFO detection and interpretation (Bénar et al., 2010; Park and Hong, 2019).

Nevertheless, some HFO properties have been shown to be valuable, as studies have demonstrated that HFO rates are often higher in SOZ channels across different lesion types (Jacobs et al., 2009). In addition, other features such as peak frequency (Cimbalnik et al., 2018), pulse amplitude (Karpychev et al., 2022), duration (Staba et al., 2002), and phase-amplitude coupling (Amiri et al., 2016) or frequency-dependent entropy (Sato et al., 2019) have also been explored as potential SOZ biomarkers.

Recently, it has been observed that HFOs can exhibit consistent propagation patterns, suggesting that brain connectivity may play a role in their generation. This is consistent with the idea of epilepsy as a “network disease” (Jehi, 2018; Li Hegner et al., 2018), e.g., even focal seizures can introduce disturbances in connectivity in large brain regions (Zentner, 2020, p. 384). Consequently, a measure of connectivity in the ripple band (80–200 Hz) has been used to identify brain regions to target for resection during epilepsy surgery (Fedele et al., 2017). Connectivity in interictal periods has also been used to predict of the efficacy of a potential resection area, with efficiency varying by frequency band (Shah et al., 2019).

**FIGURE 1:**
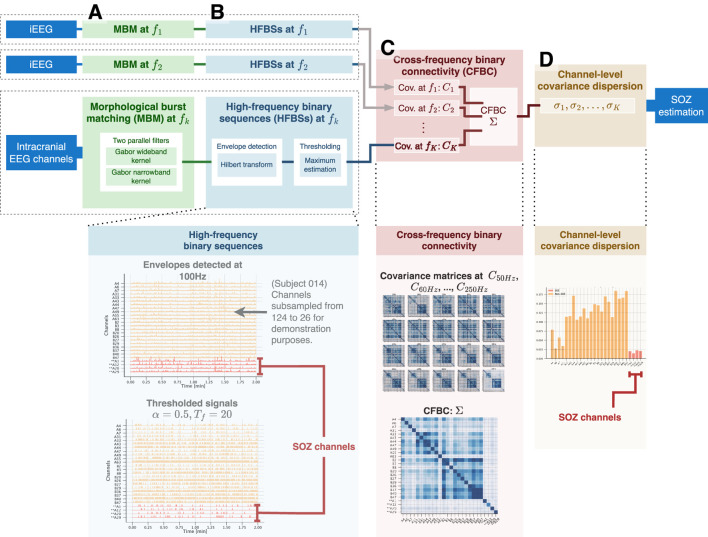
Calculation process of the channel-level covariance dispersion (CLCD) metric. For each iEEG channel, **(A)** the morphological burst matching (MBM) is applied in the target frequency $f_{k}$ , which involves two parallel filters working with different bandwidths to mitigate the impact of wideband artifacts. Then, **(B)** we use a Hilbert transform to estimate the envelope of the filtered signal. The envelope is then followed by a thresholding method to generate a binary sequence known as the high-frequency binary sequence (HFBS). **(C)** Next, we estimated the covariance matrix for the HBFS sequences at frequency $f_{k}$ over all iEEG channels. Subsequently, we collect the covariances from all frequencies of interest $f_{1}$ , $f_{2}$ , … $f_{k}$ . We combine all of those matrices into a cross-frequency binary connectivity (CFBC) matrix. **(D)** Finally, we calculate the CLCD score from the CFBC matrix. Note that HFBS sequences describe burst pulses occurring at a particular frequency that is less susceptible to be generated by artifacts (with spectrum in the same frequency band). Therefore, HFBS rates may differ from HFO rates as described in the literature.

This paper proposes a method for measuring electrode synchronization in high frequencies as a candidate biomarker of SOZ channels (Figure 1). First, we identify high-frequency bursts in the ripple band (80–200 Hz) that follow the morphological definition of an HFO. We enhance this search by including a two-step filter to minimize the effect of artifacts, attenuate pulses with short durations, and filter the signal within narrow frequency bands. Then, we combined connectivity between electrodes across different frequencies into a single connectivity matrix. We use as a biomarker the dispersion of the connectivity values observed at each channel. We validated our procedure in two open-access datasets with sampling frequencies of approximately 1000 Hz. Abnormal synchronization patterns observed through this biomarker are associated with the SOZ.

## 2 Methods

### 2.1 Gabor transform as a HFO-like kernel

Definitions of HFOs differ in the literature, and when evaluating the results, the exact frequency interval, filter settings, and identification method need to be taken into consideration. (Zijlmans et al., 2017). However, we can generally consider an HFO to be a transient burst from which at least a finite number of consecutive cycles are readily distinguished from the surrounding background. When simulating these oscillations, they are often modeled as the result of multiplying a sine wave with a Gaussian envelope (Donos et al., 2020):

$$h^{*}\left(t;f_{0},\sigma \right)=g\left(t,\sigma \right)\sin\left(2\pi f_{0}t\right)$$ (1)

where $g\left(t,\sigma \right)$ is a Gaussian envelope with its shape controlled through a dispersion parameter σ such that $g\left(t,\sigma \right)=\exp\left(-\frac{1}{2}{\left(\frac{t}{\sigma }\right)}^{2}\right)$ .

A notable property of this model (Equation 1) is its potential for extension by considering that the oscillatory component is a complex value:

$$h\left(t;f_{0},\sigma \right)=g\left(t,\sigma \right)\exp\left(j2\pi f_{0}t\right)$$ (2)

With this notation, a high-frequency burst $h\left(t;f_{0},\sigma \right)$ is also the kernel of a Gabor, or Morlet, transform. Then, the kernel function of a Gabor transform can be used to model and isolate high-frequency bursts that fit the definition of an HFO. This interpretation allows us to extrapolate several properties from high-frequency bursts. For instance, by convolving the signal with a Gabor kernel of frequency $f_{0}$ , we recover the time τ of any burst with the same frequency, $h\left(t-\tau ;f_{0},\sigma \right)$ , while bursts at other frequencies will be attenuated.

In this paper, we propose to use the following scaled Gabor kernel:

$$h\left(t;f_{0},\sigma \right)=\frac{1}{\sqrt{2\pi \sigma^{2}}}g\left(t,\sigma \right)\exp\left(j2\pi f_{0}t\right)$$ (3)

This formulation ensures time and frequency properties (with closed-form solutions) that allow us to select an optimal dispersion σ . Formal descriptions and proofs of the Gabor kernel (Equations 2, 3) properties are included in Supplementary Appendix A. A summary of the main properties involving the Gabor dispersion is shown in Table 1.

**TABLE 1:** Design parameters for a Gabor kernel filter. The approximated expressions are determined under the following assumptions: a) the bandwidth is defined as the interval where the spectrum is equal to or greater than half of its maximum value, b) the amplitude in the filtered pulse is equal to or greater than 98.8% of the unfiltered amplitude, c) the transient duration is defined as the interval over which the signal exceeds 25% of its filtered maximum amplitude. Further information on these expressions is found in Supplementary Appendix A1.

Parameter	Approximated expression
Frequency bandwidth	$\Delta f\approx 0.1874\;\sigma^{-1}$
Minimum duration without attenuation	$B^{*}\approx 5\sigma$
Relative transient duration for large pulses	Δt≈0.674σ
Relative transient duration for short pulses	Δt≥1.665σ

### 2.2 Gabor kernel as a frequency and pulse filter

We can configure the Gabor kernel to attenuate pulses with a lower duration than desired, thus complying with the number of oscillations required by the HFO definition. Let C be the number of cycles at a frequency $f_{0}$ that we assume is the minimum to be considered a valid pulse. Then, let B be the pulse width such that $B=\frac{C}{f_{0}}$ .

Without loss of generality, assume a unit input amplitude. Then a Gabor kernel with dispersion σ will ensure an output amplitude equal to or higher than 0.988 for a pulse duration equal to or higher than 5σ . For alternative levels of output amplitude, the equations are provided in Supplementary Appendix A2. A similar dispersion-controlling procedure was suggested in (Waldman et al., 2018) where $\frac{f_{0}}{\sigma }=7$ is recommended without further rationale.

We should note that Gabor dispersion σ also has an impact on the filter frequency response, where a larger dispersion implies a narrower filter in the frequency domain. Let us define the frequency bandwidth Δf as the range of frequencies within which the filter has a gain equal to or greater than a specified cutoff gain $\eta_{f}$ . Typically $\eta_{f}=\frac{1}{2}$ in filter design applications (Semmlow, 2004, pp. 14–15). Subject to this cutoff, the frequency bandwidth is described by

$$\Delta f={\left(2\pi \sigma \right)}^{-}1\sqrt{-2\log\eta_{f}}\approx \frac{0.588705}{2\pi \sigma }$$ (4)

Proof of this approximation is included in Supplementary Appendix A1.

### 2.3 Morphological burst matching (MBM)

Artifacts and other iEEG events, such as epileptiform discharges, DC shifts, and sharp transients, have a broadband spectrum that spans to high frequencies (Bénar et al., 2010). Due to their broad frequency ranges, these signals are sometimes identified as “candle”-shaped in the time-frequency decomposition (Waldman et al., 2018). Some of them have a spectrum in the range 2–120 Hz (Sanei and Chambers, 2007, p.26) with the potential to overlap with the ripple band, 80–200 Hz. In contrast, true HFOs tend to have power concentrated around specific frequencies (morphologically described as “blobs” or “islands”) (Waldman et al., 2018).

**FIGURE 2:**
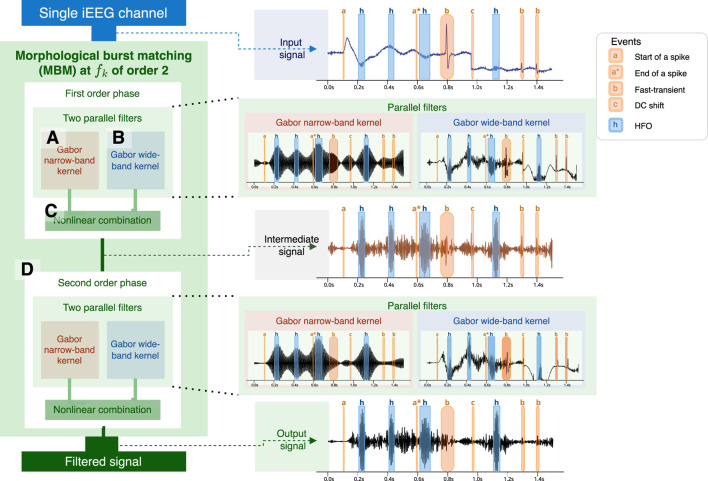
Calculation process of a second-order MBM filter. The first step involves concurrently using two filters: one narrowband filter **(A)** based on the number of oscillations required for an HFO and a wideband filter **(B)**. Envelopes are estimated from both signals and then merged. Merging **(C)** is performed by multiplying by the phase of the wideband-filtered envelope and the amplitude narrowband-filtered envelope. The process may be iterated several times **(D)**, with the number of iterations referred to as the filter order. At the right, we show an example of the application of the MBM filtered using a simulated signal. HFO are highlighted in blue with a label “h”. Artifacts are highlighted in red: start of a spike and slow wave, or spike (“a”), end of the spike (“a*“), fast-transient (“b”), DC shift (“c”).

An MBM filter uses two parallel filters in an iterative process to mitigate broadband oscillations while highlighting narrowband oscillations (Figure 2). It starts by constructing two Gabor filters (Equation 3) with the same central frequency $f_{0}$ . The first, a narrowband (NB) filter, is configured with a dispersion $\sigma^{-}$ such that it attenuates pulses with fewer cycles than our goal. The second is a wideband (WB) filter, controlled with a dispersion $\sigma^{+}$ that intends to resemble the original signal while capturing the bandwidth, where we still expect to see the effect of the artifact.

Then, we create a new envelope (instantaneous amplitude) by taking the minimum value at each time point t between the envelopes of the filtered signals:

$$A_{\text{common}}\left(t\right)=\min\left\{A\left\{x_{f_{0}}^{-}\left(t\right)\right\},A\left\{x_{f_{0}}^{+}\left(t\right)\right\}\right\}$$ (5)

where $x^{-}\left(t\right)=h\left(t;f_{0},\sigma^{-}\right)*x\left(t\right)$ , $x^{+}\left(t\right)=h\left(t;f_{0},\sigma^{+}\right)*x\left(t\right)$ , and $A\left\{x_{f_{0}}^{\cdot }\left(t\right)\right\}$ is the instantaneous amplitude of the analytic signal of $x_{f_{0}}^{\cdot }\left(t\right)\left(t\right)$ .

Finally, we can assemble the filtered signal as the combination of the new envelope (Equation 5) and the instantaneous phase of the NB filtered signal:

$$x_{f_{0}}\left(t\right)=A_{\text{common}}\left(t\right)\exp\left(jP\left\{x_{f_{0}}^{-}\left(t\right)\right\}\right)$$ (6)

where $P\left\{x_{f_{0}}^{-}\left(t\right)\right\}$ is the instantaneous phase of the analytic signal of $x_{f_{0}}^{-}\left(t\right)\left(t\right)$ .

This process can be iterated several times by using the output of the previous iteration as the next input. The total number of iterations is named as the filter order.

### 2.4 Binary sequence reflecting burst times

Let Y(t) be the multivariate time series that contains the iEEG signals of P channels: $Y\left(t\right)=\left\{y_{1}\left(t\right),y_{2}\left(t\right),\ldots ,y_{P}\left(t\right)\right\}$ . To generate a set of binary sequences for a channel k , we start by calculating the envelope (instantaneous amplitude) of a filtered signal $x_{f_{0}}\left(t\right)$ around a frequency $f_{0}$ (Equation 6):

$$A_{Y_{k},f_{0}}\left(t\right)=A\left\{x_{f_{0}}\left(t\right)\right\}$$ (7)

Then, a threshold $\tau_{k}$ is defined as an estimate of the maximum value $M_{k,f_{0}}$ such that $\tau_{k}=\alpha_{\tau }M_{k,f_{0}}$ , with the factor α setting the strictness of the threshold. We propose using an estimation of the maximum reference value across fixed non-overlapping intervals of $T_{\tau }$ time points:

$$M_{k,f_{0}}=E\left[\max\left({\left\{A_{Y,f_{0}}\left(t+\nu \right)\right\}}_{\nu =0}^{\nu =T_{\tau }}\right.\right]$$ (8)

The advantage of using this estimator is its ability to mitigate the impact of extreme values observed due to artifacts that were not attenuated with the MBM filter.

This threshold value is then used to create a high-frequency binarized sequence (HFBS), $B_{k,f_{0}}\left(t\right)$ , for the k -th channel. The envelope $A_{Y_{k},f_{0}}$ is (Equation 7) binarized using a threshold $\tau_{k}$ (estimated with Equation 8):

$$B_{k,f_{0}}\left(t\right)=I\left[A_{Y_{k},f_{0}}\left(t\right)\geq \tau_{k}\right]$$ (9)

where I[Θ] is the indicator function that returns 1 only when Θ is true.

### 2.5 Cross-frequency binary connectivity

The HFBS signals for P channels filtered at a frequency $f_{0}$ constitute a multivariate time series $B_{f_{0}}\left(t\right)=\left\{B_{1,f_{0}}\left(t\right),B_{2,f_{0}}\left(t\right),\ldots ,B_{P,f_{0}}\left(t\right)\right\}$ . Our approach involves using a covariance matrix as a functional connectivity metric (Aqil et al., 2020; Razi and Friston, 2016) on the binarized signal (Equation 9), defined as $C_{f_{0}}=\left[B_{f_{0}}^{T}\left(t\right)B_{f_{0}}\left(t\right)\right]$ . In this context, covariance provides a measure of synchronization between the binarized pulses (Ombao and Pinto, 2022).

The MBM filter and cross-frequency binary connectivity are designed to operate on single frequencies with an inherent frequency tolerance. Therefore, we gathered covariance matrices for a specific set of F frequencies $\Theta_{f}=f_{1},f_{2},\ldots ,f_{F}$ to quantify the variation in a specific range of frequencies.

To represent an aggregate connectivity matrix across the $\Theta_{f}$ frequencies, we assign a weight $w\left(C_{f_{i}}\right)$ to each matrix $C_{f_{i}}$ , such that we can create a cross-frequency covariance matrix as a weighted average:

$$\Sigma =\overset{F}{\underset{i=1}{\sum }}w\left(C_{f_{i}}\right)C_{f_{i}}$$ (10)

This formulation allows us flexibility in the strategy to combine the connectivity matrices. If we assume that all covariance matrices have the same impact, the weight should be constant $w\left(C_{f_{i}}\right)=1$ for all matrices. However, we can also use the weight to minimize the impact of matrices containing outliers, such as abnormal values or sparsity. A simple metric that can be used to quantify this is the standard deviation of the items in such matrices. Therefore, we suggest using the weight $w\left(C_{f_{i}}\right)=\frac{1}{\text{std}\left(C_{f_{i}}\right)}$ that penalizes matrices with those outliers. We should remark that this weighting rule will only attenuate abnormal values that happen in some, but not all, frequencies.

### 2.6 Channel-level covariance dispersion (CLCD)

The combined connectivity matrix Σ (Equation 10) provides information about the synchronization between each pair of channels i and j through the $\sigma_{i,j}$ . Thus, for each channel k , the vector of covariances $\left\{\sigma_{k,1},\sigma_{k,2},\ldots ,\sigma_{k,P}\right\}$ quantifies its relationship with every other electrode. We can summarize this vector by measuring its dispersion using the sample standard deviation of its values:

$${\tilde{\eta }}_{k}=\frac{1}{P-1}\overset{P}{\underset{m=1}{\sum }}{\left(\sigma_{k,m}-\frac{1}{P}\overset{P}{\underset{i=1}{\sum }}\sigma_{k,i}\right)}^{2}=\text{std}\left(\left\{\sigma_{k,1},\sigma_{k,2},\ldots ,\sigma_{k,P}\right\}\right)$$ (11)

This metric quantifies the variability in connectivity that channel k experiences in the system. Thus, a channel with similar connectivity to all other channels will have a lower score than channels that have different connectivity values with a specific subgroup of electrodes. We hypothesized that the SOZ channels would exhibit anomalous connectivity values compared to the non-SOZ (nSOZ) channels, which could be reflected as abnormal CLCD values.

Then, we defined the channel-level connectivity dispersion (CLCD) as the z-score of the metric ${\hat{\eta }}_{k}$ (Equation 11):

$$n_{k}=\frac{{\tilde{\eta }}_{k}-\sum_{m=1}^{P}{\tilde{\eta }}_{m}}{\text{std}\left(\left\{{\tilde{\eta }}_{1},{\tilde{\eta }}_{2},\ldots ,{\tilde{\eta }}_{P}\right\}\right)}$$ (12)

## 3 Dataset

To evaluate CLCD scores as a candidate marker of the SOZ, we use two open-access datasets that contains iEEG recordings from patients with drug-resistant focal epilepsy. The first dataset, CHM, was collected at Children’s Hospital of Michigan and Harper University Hospital, Detroit Medical Center (Sakakura and Eishi Asano, 2023; Sakakura et al., 2023). Recordings from these patients were collected at 1000 Hz during 20 min of slow-wave sleep in interictal periods separated at least 2 hours from any clinical-determined seizure event. All patients in this dataset had an International League Against Epilepsy (ILAE) class 1 outcome in their last follow-up, implying that they were considered seizure-free without the presence of any auras. We refer to (Sakakura et al., 2023) for further details on the experimental setup, inclusion, and exclusion criteria.

Within this group of 114 patients, we focused on those who had more than two SOZ channels in subdural grid electrodes. We use these inclusion criteria because CLCD is a relative statistic that relies on the values of all electrodes. Consequently, a low or nonexistent number of SOZ channels might significantly distort our results. The selected subset comprised 89 patients with an average age of 10.90 years (standard deviation: 5.73), of which 47.2% were female. 65.2% had a visible MRI lesion. SOZ locations were primarily identified in the temporal (49.4%), parietal (31.5%), frontal (30.3%), and occipital (20.2%) regions.

We also used a second open-access dataset, HUP, collected at the Hospital of the University of Pennsylvania (Bernabei et al., 2023a; b, 2022). This dataset consists of ictal and interictal recordings from 85 patients. Each patient had implanted subdural or depth electrodes or a combination of both. Resection or laser ablation was used as a surgical procedure. Surgery outcome was evaluated using the Engel scale at least 6 months after the intervention. We refer to (Bernabei et al., 2023b; Bernabei et al., 2022) for additional details on the experimental setup.

The recordings in this dataset had frequency samplings between 500 Hz and 1024 Hz. To match the sampling frequency of the HUP dataset, we selected only the subjects that had at least 1000 Hz as the sampling rate with at least one identified SOZ channel. Then, the subset used in our study comprised 15 patients with an average age of 31.87 years (standard deviation: 9.76), of which 53.3% were female. 33.3% had a visible MRI lesion, and 33.3% had a resection procedure. Moreover, the patient outcomes reported were: Engel I-A (n = 4), I-B (n = 4), I-D (n = 2), III-A (n = 4), IV-A (n = 1). Furthermore, 14 patients had implanted depth electrodes, and only a single patient had a subdural electrode. Moreover, the electrodes were mainly placed in the medial temporal lobe (60%) and temporal lobe (20%).

For each subject, we selected three non-overlapped 2-min epochs of iEEG for our analysis, and we chose the frequency singletons in the ripple band (80–200 Hz). Each MBM filter was configured to penalize pulses of bursts with less than five oscillations in each frequency, while the wideband filter was designed to have a bandwidth of 25 Hz around each filtering frequency.

## 4 Results

### 4.1 MBM filter comparison

**FIGURE 3:**
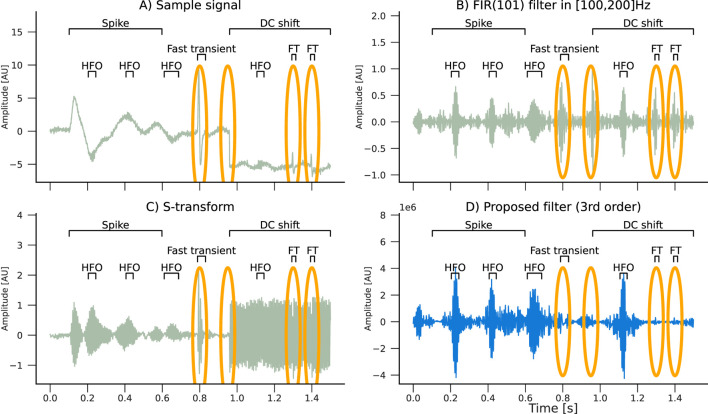
Filtering effects of an **(B)** FIR(101), an **(C)** S-transform, and an **(D)** MBM filter with a **(A)** simulated signal composed of HFO pulses with a spike and slow wave (spike), three fast transients (FTs), and a DC shift. Artifacts are highlighted in orange ellipses. Only the real component of the S-transform is plotted. Note that the MBM filter automatically attenuated the artifacts.

We simulated an EEG signal composed of a spike, three fast transients, and a DC shift, along with a set of HFO oscillations with different pulse widths at a central frequency of 150 Hz (Figure 3A). Then we applied two filters: a 101-th-order FIR filter with a bandwidth of 100 Hz and an S-transform kernel used as a filter. In addition, we applied a third order MBM filter. The filtering results are compared in Figures 3.B–D.

Furthermore, we assessed the capability of the filter to reconstruct the original high-frequency pulse in the presence of artifacts. We simulated three scenarios, each one denoting an HFO of 50 ms duration with a unit amplitude followed by either a spike, a short sharp transient (1 ms), or a fast sharp transient (3 ms). These scenarios were designed to represent the influence of artifacts with a variable signal-to-noise ratio in comparison to the true HFO pulse. Simulations were performed using a sampling frequency of 1,000 Hz. We considered two HFO frequencies: 100 Hz and 200 Hz. The filters’ responses were assessed for artifacts with amplitudes ranging from 0 (no artifact) to 10. Thus, 264 combinations were evaluated, in total.

**FIGURE 4:**
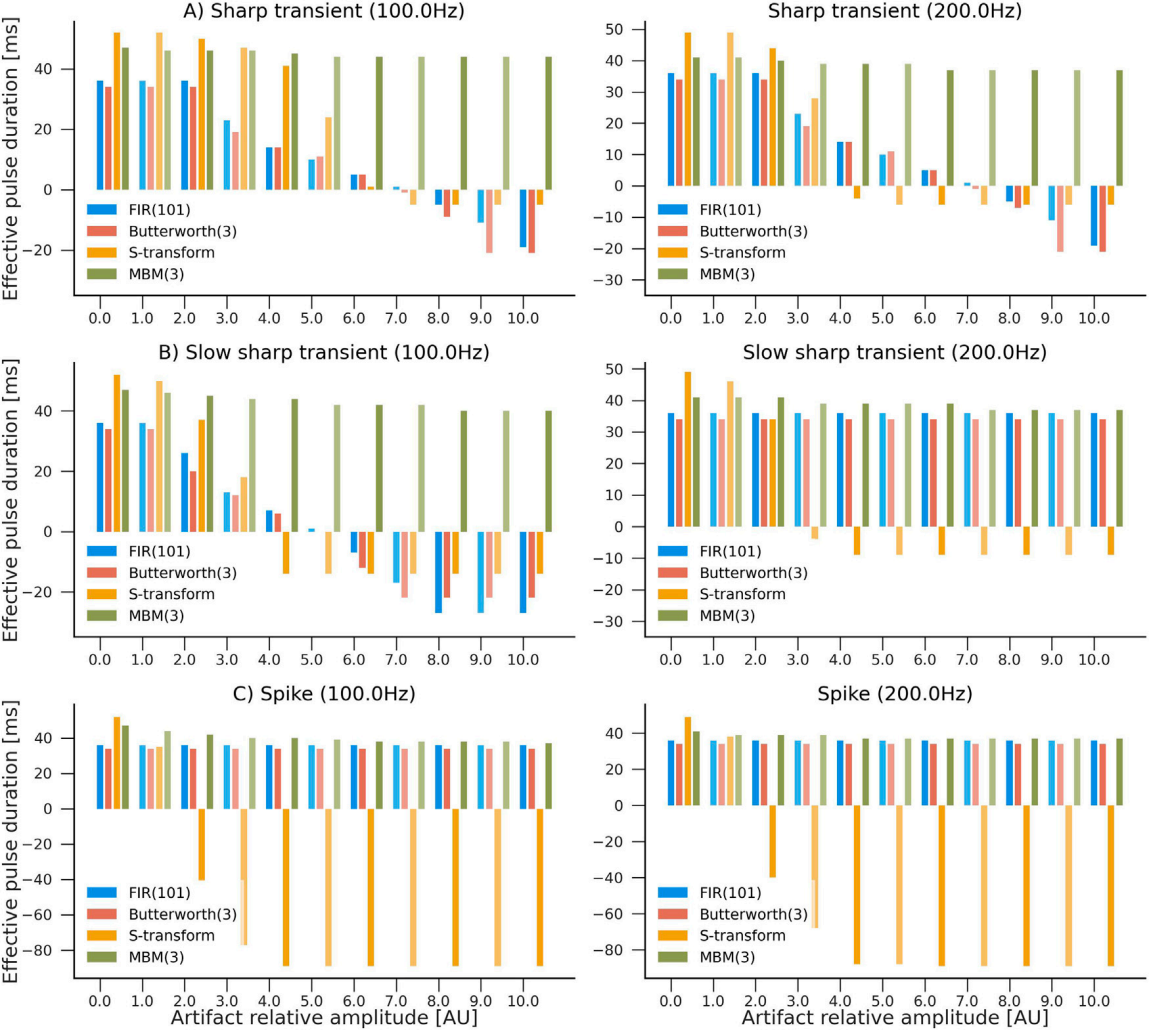
Evaluation of the influence of artifacts in pulse reconstruction after filtering. Higher values indicate higher accuracy in reconstructing HFO-like events in the presence of an artifact. The simulated signal consisted of a single HFO pulse lasting 50 ms, followed by an artifact: **(A)** sharp transient, **(B)** slow sharp transient, or **(C)** a spike with frequencies of 100 Hz and 200 Hz. Four filters were compared: S-transform, FIR(101), Butterworth (third order), and MBM (third order). Artifacts’ amplitudes ranged from 0 (no artifact) to 10 times the amplitude of the HFO pulse. Y-axis represents the “effective pulse duration” in milliseconds. This number is closer to the true pulse length (50 ms) when there are no artifacts present. However, it becomes negative when the duration of artifact-caused pulses exceeds the estimated duration of the actual pulses.

The estimated pulses were calculated using the binarization procedure outlined in Section 2.4. In order to evaluate the filter’s performance, we calculate the “effective total duration,” in which correctly identified pulse durations were multiplied by a factor of +1, while incorrectly identified pulse durations were mutiplied by a factor of −1. Consequently, the effective duration turns negative when the duration of false pulses exceeds that of the actual pulses. An overview of the simulation results is shown in Figure 4.

Typical spikes, representative of epileptiform discharges, had minimal influence on all filters except the S-transform, where the impact was more noticeable. Nevertheless, abrupt transients considerably impacted all filters except MBM, which demonstrated remarkable stability against both classes of artifacts in the simulations. This robustness of the MBM filter is a significant finding of our study.

### 4.2 Separability

**FIGURE 5:**
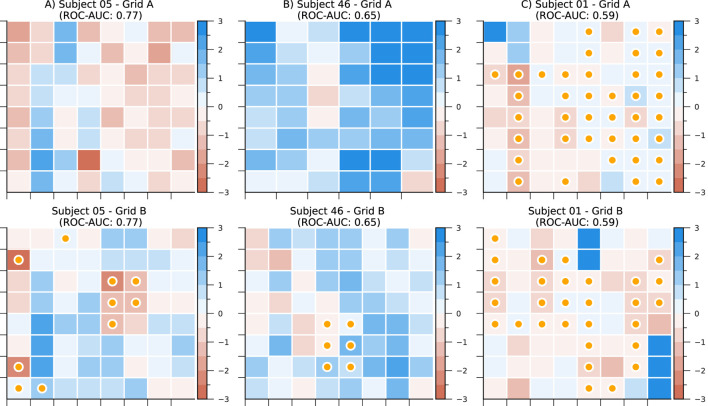
CLCD scores in subdural grids from subjects **(A)** CHM05, **(B)** CHM46, and **(C)** CHM01. Darker colors represent lower scores. Channels labeled as SOZ are marked with a yellow bullet. ROC-AUC as a separability metric is also included for each electrode grid.

Figure 5 illustrates the application of our CLCD scores in identifying potential SOZ channels within grids of subdural electrodes. A CLCD score is associated with every channel based on its connectivity dynamics, as described in Section 2.6. We hypothesized that extreme values would be associated with the SOZ channels. Thus, in the rest of this section, we apply several performance evaluation techniques to determine the separation between the CLCD scores observed in SOZ and nSOZ channels.

**FIGURE 6:**
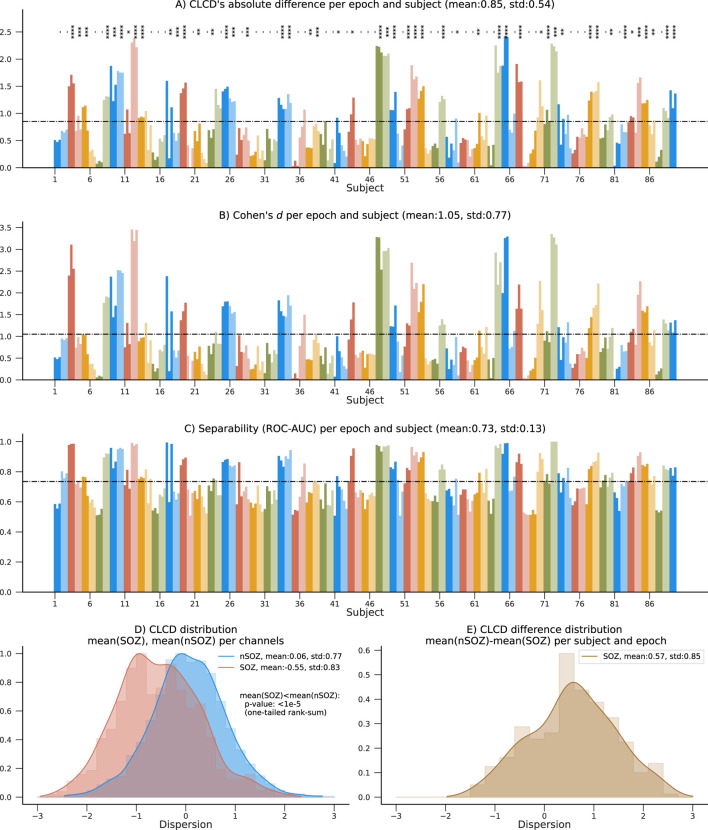
Summary results on the 89 patients in the CHM dataset. **(A)** Absolute difference between average nSOZ-CLCD and SOZ-CLCD for each subject and epoch. Statistical difference is assessed using a rank-sum test (*p*-values:*: < 0.05, **: < 0.01, ***: < 0.001, ****: < 0.0001). **(B–C)** Measures of separability between SOZ and nSOZ channels are shown: Cohen’s d and ROC-AUC. **(D)** Distribution of CLCD values in SOZ and nSOZ channels. The median of the nSOZ channels is statistically significantly higher than in SOZ channels (rank-sum test, *p*-value < 1e-5). **(E)** Distribution of the mean CLCD in nSOZ channels subtracted from the mean in SOZ channels, for all epochs and subjects.

First, we calculated the CLCD metric for the iEEG data from each of the 89 patients with refractory epilepsy in the CHM dataset and 15 patients in the HUP dataset. We should recall that the iEEG signals were sampled at approximately 1000 Hz (CHM dataset: 1000 Hz, HUP dataset: 1000 Hz and 1024 Hz) and, consequently, the CLCD metric was calculated on the ripple band (80–200 Hz) as described in Section 3. For each subject, we calculated the CLCD associated with the SOZ channels (SOZ-CLCD) and nSOZ channels (nSOZ-CLCD). The absolute value of the difference between the average SOZ-CLCD and the average nSOZ-CLCD for each subject and epoch is depicted in Figure 6A (CHM dataset) and 7. A (HUP dataset. We found an average of 0.85 and standard deviation of 0.54 (CHM) and an average of 0.86 with an standard deviation of 0.36 (HUP), indicating a notable difference between CLCD in SOZ and nSOZ channels. We also evaluated the significance of the difference between SOZ-CLCD and nSOZ-CLCD for each subject using the Wilcoxon-Mann-Whitney, or rank-sum, test. *p*-values for each subject were adjusted for multiple comparisons using the Benjamini-Yekutieli procedure. From this procedure, we found that forty-nine subjects (55.06%) and thirteen subjects (86.66%) exhibited a significant difference (*p*-value < 0.05) in the CHM and HUP datasets, respectively.

**FIGURE 7:**
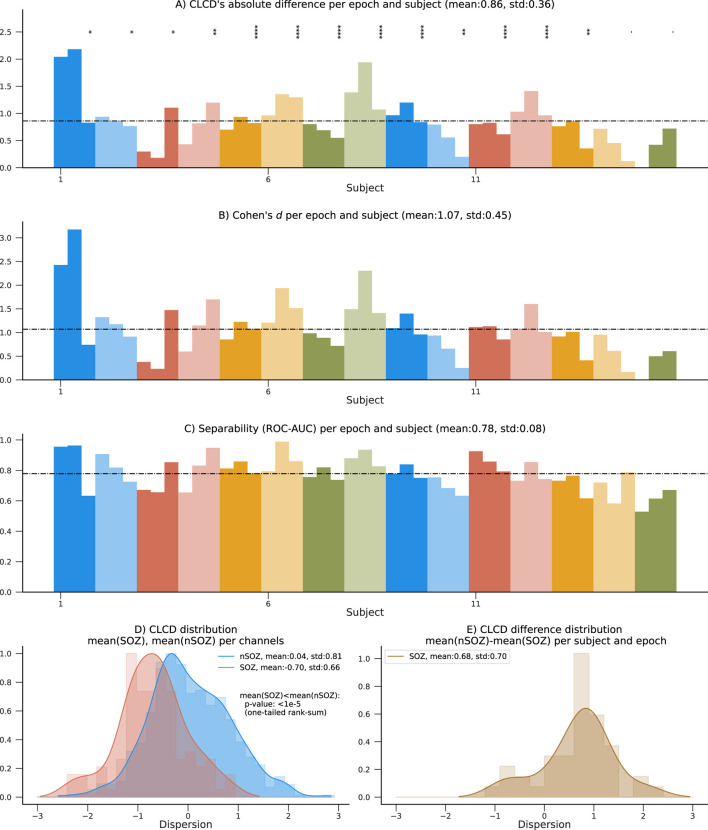
Performance results on the 15 patients in the HUP dataset using SOZ channels. **(A)** Absolute difference between average nSOZ-CLCD and SOZ-CLCD for each subject and epoch. Statistical difference is assessed using a rank-sum test (*p*-values:*: < 0.05, **: < 0.01, ***: < 0.001, ****: < 0.0001). **(B–C)** Measures of separability between SOZ and nSOZ channels are shown: Cohen’s d and ROC-AUC. **(D)** Distribution of CLCD values in SOZ and nSOZ channels. The median of the nSOZ channels is statistically significantly higher than in SOZ channels (rank-sum test, *p*-value < 1e-5). **(E)** Distribution of the mean CLCD in nSOZ channels subtracted from the mean in SOZ channels, for all epochs and subjects.

To measure our method’s ability to classify SOZ and nSOZ channels, we used two separability metrics. First, we calculated Cohen’s d, often used for effect size analysis, which also provides information about the amount of overlap between the SOZ-CLCD and nSOZ-CLCD. Across subjects, the average Cohen’s d was 1.05 (standard deviation: 0.77, Figure 6B) and 1.07 (standard deviation: 0.7457, Figure 7B) in the CHM and HUP datasets, respectively.

Finally, we estimated the concordant statistic (c-statistic) through the area under the receiver operating characteristic curve (ROC-AUC). This metric assessed the CLCD’s capability to provide a threshold that isolated SOZ clusters (Hand and Till, 2001). Results for each subject are in Figure 6C; patients showed an average ROC-AUC of 0.73 (standard deviation:0.13) in the CHM dataset and an average ROC-AUC of 0.78 (standard deviation: 0.08) in the HUP dataset.

### 4.3 CLCD magnitude in SOZ channels

The distribution of SOZ-CLCD and nSOZ-CLCD values across the channels in all subjects is depicted in Figure 6D. The nSOZ channels had an average CLCD of 0.059 (standard deviation: 0.774). In contrast, SOZ channels averaged −0.550 (standard deviation: 0.826). In our dataset, CLCD values in SOZ were statistically significantly lower than nSOZ channels (one-tailed rank-sum test, *p*-value < 1e-5).

As expected, the distribution of the difference between nSOZ-CLCD and SOZ-CLCD per subject and epoch (Figure 6E) was positively skewed with an average of 0.570 and a standard deviation of 0.851.

### 4.4 Separability and CLCD magnitude in resected channels

**FIGURE 8:**
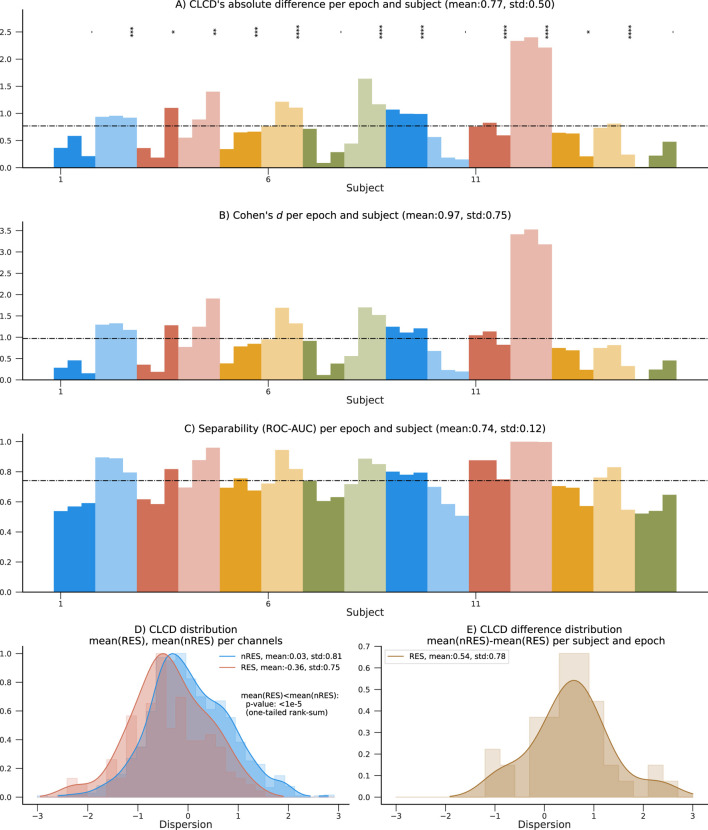
Performance results on the 15 patients in the HUP dataset using resected channels. **(A)** Absolute difference between average nRES-CLCD and RES-CLCD for each subject and epoch. Statistical difference is assessed using a rank-sum test (*p*-values:*: < 0.05, **: < 0.01, ***: < 0.001, ****: < 0.0001). **(B–C)** Measures of separability between RES and nRES channels are shown: Cohen’s d and ROC-AUC. **(D)** Distribution of CLCD values in RES and nRES channels. The median of the nRES channels is statistically significantly higher than in RES channels (rank-sum test, *p*-value < 1e-5). **(E)** Distribution of the mean CLCD in nRES channels subtracted from the mean in RES channels, for all epochs and subjects.

The 15 patients in the HUP dataset used for validation also contain information about resected (RES) and non-resected (nRES) channels. Similar to the performance evaluation procedure described in Section 4.2, we estimated the CLCD associated with the resected channels (RES-CLCD) and the non-resected channels (nRES-CLCD). Our method still denotes differences between RES-CLCD and nRES-CLCD, and the pattern is similar to the values observed when the CLCD values were compared against the SOZ labels (Figures 7.A–C). The absolute value for each subject and epoch has an average of 0.77 and a standard deviation of 0.50 (Figure 8A). Cohen’s d values had an average of 0.97 with a standard deviation of 0.75 (Figure 8B). Moreover, the concordant statistic or ROC-AUC is 0.74 with a standard deviation of 0.12.

While the values in the non-resected channels are still statistically significantly higher than in non-resected channels (*p*-value < 1e-5, one-tailed rank-sum, Figure 8D), the magnitude of the difference is considerably lower than when we only analyze SOZ channels (Figure 7D). This disparity may have arisen due to the inclusion of resected channels that were in non-seizure-generating brain regions and the exclusion of previously channels classified as SOZ, which could have led to supplementary clinical assessments during surgery that were not reported in the validation dataset. This discrepancy could explain the performance contrast in subject HUP01, which shows a difference in the CLCD values in SOZ and nSOZ channels but poor differentiation between CLCD in RES and nRES. The opposite is true in subject HUP12, where the CLCD values perform better at separating RES/nRES channels (Figures 8.A–C).

### 4.5 CLCD and surgical outcome prediction

**FIGURE 9:**
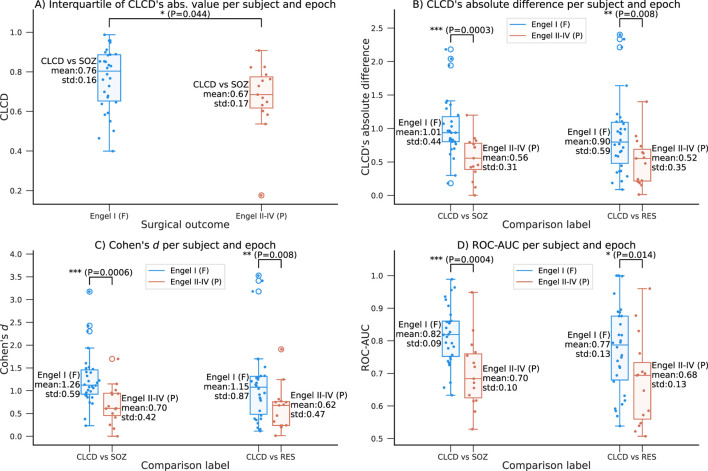
Relationship between the CLCD values and the outcome Engel scale in the HUP dataset. **(A)** Interquartile range of the absolute CLCD values as a function of the surgical outcome. A favorable outcome (Engel I) is associated with larger range of CLCD magnitudes. **(B–D)** Variation of the separability metrics with respect to the Engel scale as described in Figures 7, 8 comparing SOZ-CLCD and nSOZ-CLCD (left) and RES-CLCD and nRES-CLCD (right): **(B)** Absolute difference for each subject and epoch, **(C)** Cohen’s d and **(D)** ROC-AUC. Mean and standard deviation are shown along each boxplot. Statistical differences are assessed using a one-tailed rank-sum test (*p*-values:*: < 0.05, **: < 0.01, ***: < 0.001, ****: < 0.0001). Note that in all cases, the higher the separability, the higher the likelihood of a favorable outcome.

We also evaluate the predictive capability of the CLCD scores in determining the surgical outcome. Note that by construction, the CLCD scores inside a grid have a zero mean and a unit standard deviation (Equation 12). Therefore, to aggregate all the values per epoch per subject, we use the interquartile range (IQR), which measures the spread of the observed CLCD values and it may be a representative metric for comparing results between different subjects. In addition, in order to mitigate the impact of outliers, we calculate the z-score of the absolute CLCD values (at each epoch), and we exclude any values where the z-score was above +3 or below −3. The IQR was computed for this subset of filtered values for each grid (Figure 9A). The patients with favorable outcomes (F: Engel I) had an interquartile average of 0.76 (standard deviation: 0.16), whereas the participants with poor outcomes (P: Engel II-IV) had an average of 0.67 (standard deviation: 0.17). Statistical significance was assessed using a one-tailed rank-sum test (*p*-value: 0.044).

Additionally, we explore the relationship between the performance of the CLCD score as an SOZ biomarker and the Engel scale. We conducted the analysis on the same evaluation metrics described in Section 4.2, where we evaluated the separability of CLCD-SOZ and CLCD-nSOZ values. We observed a difference in the CLCD’s absolute difference per subject and epoch (F: 1.01, P: 0.56, *p*-value: 6e-4), in the Cohen’s d values (F: 1.26, P: 0.70, *p*-value: 1e-3), and in the separability, ROC-AUC (F: 0.82, P: 0.70, *p*-value = 2e-4) values. As shown in Figure 9C, D, all performance metrics consistently showed higher CLCD separability magnitudes in patients with favorable outcomes compared to those who demonstrated poor outcomes.

Our previous scores showed some difference in the performance results when the resected channels were used instead of the SOZ labels. We analyzed the association of the performance of CLCD-RES and CLCD-nRES as a function of the Engel scores. We found that performance metrics are still significantly higher in patients with favorable outcomes (Figure 9C, D): in CLCD’s absolute differences (F: 0.90, P: 0.52, *p*-value = 8e-3); in the Cohen’s d values (F: 1.15, P: 0.62, *p*-value = 8e-3) and in the ROC-AUC (F: 0.77, P: 0.68, *p*-value = 0.014).

## 5 Discussion

This paper proposes a method that uses connectivity information across burst-associated binary sequences at different frequencies to generate a dispersion score (CLCD) for each channel. We hypothesized that this score could assist in delineating the SOZ.

As part of our approach, we developed an enhanced filter, MBM. This filter is based on a Gabor kernel function and was used prior to generating the binary sequences. Our mathematical proofs and experimental simulations demonstrate that the MBM filter can successfully attenuate specific categories of artifacts with large spectral bandwidths, including spikes, sharp transient events, and DC shifts, which are often challenging to remove using only frequency-filtering methods. It is essential to acknowledge that our MBM filter has limitations. For instance, additional simulations are required to validate its efficacy in removing muscle artifacts, since they were not included in our initial analysis. Moreover, by construction, any waveform with a spectrum overlapping our central frequency could be significantly attenuated if its spectrum bandwidth is broader than the bandwidth configured in the MBM filter. Suppose a physiological event produces two bursts simultaneously, one in the ripple band and another in the fast ripple band. In such a circumstance, an MBM filter will attenuate both signals as they can be misinterpreted as broadband artifacts. Nevertheless, with its constraints, an MBM filter minimizes the likelihood of DC shifts and spikes affecting our connectivity results, as they have been shown to be sources of artifactual bursts in high frequencies (Lee et al., 2020). Our MBM filter also enhances the differences in CLCD scores between SOZ and nSOZ, when compared to FIR filters or the root-mean-square HFO detection method (Staba et al., 2002) (see Supplementary Material).

The CLCD score assesses the degree of similarity in synchronization between one electrode with respect to the other electrodes in the subdural grid. SOZ channels will have extreme values, denoting an abnormal behavior in the connectivity network, that can be used as a biomarker. Our analysis (performed in signals sampled at ∼ 1000 Hz) revealed that the CLCD score, in both datasets, estimated in the ripple band (80–200 Hz) reflects a distinct synchronization pattern within the SOZ channels, leading to a significant degree of separability, as shown by the AUC and Cohen’s d values. On the scale suggested by Hosmer and Lemeshow (Hosmer and Lemeshow, 2000, p.162), our average AUC estimate is considered “acceptable”, or satisfactory, given that it is in the range between 0.7 and 0.8. At the same time, our Cohen’s d values show that the distribution of CLCD scores in SOZ and nSOZ overlaps less than 45% (Sullivan and Feinn, 2012). This separability observed in both metrics confirms the ability of our score to classify SOZ and nSOZ channels. These results are consistent with the literature, as differences in the synchronization (cross-correlation) patterns in the ripple and fast ripple band inside and outside the SOZ (Weiss et al., 2024a) have also been reported. Although current performance assessments denote that CLCD has the potential to be used as an additional surgical tool, we recognize that it may have significant limitations as a standalone approach for surgical guidance due to its current false positive rate. However, the CLCD scores can be combined with other methodologies and clinical assessments to improve the identification of SOZ channels.

Our connectivity method can also be understood as a method to construct a weighted undirected graph. Graph theory properties have been explored in the literature as SOZ delineation biomarkers. Weiss et al. showed that local efficiency, characteristic path length, and nodal strength could provide a SOZ prediction accuracy, measured through ROC-AUC, of 0.778 between different subjects (Weiss et al., 2022), and can predict Engel scores with an ROC-AUC between 0.68 and 0.82 (Weiss et al., 2023). These studies also noted that accuracy can be improved by integrating machine learning techniques along with their graph theory-based features (Weiss et al., 2024b; 2022). Further work is required to integrate CLCD with an interpretable machine-learning method that could enhance the accuracy of the SOZ prediction.

It is important to highlight that the distribution of our CLCD scores is consistent across the CHM and HUP datasets (Figures 6.E, 7.E). Similarly, the differences in the CLCD scores in SOZ and nSOZ channels have a consistent pattern (Figures 6.D, 7.D). Given that these datasets include populations with distinct age distributions and originate from separate medical facilities, potential biases inherent to each specific dataset, such as inter-rater variability in assessing the SOZ channels that we used as a performance reference, are mitigated. Although we advise being cautious in drawing strong conclusions from these results due to the small sample size in the HUP dataset, the diverse population characteristics in both datasets speak to the robustness of our method.

**FIGURE 10:**
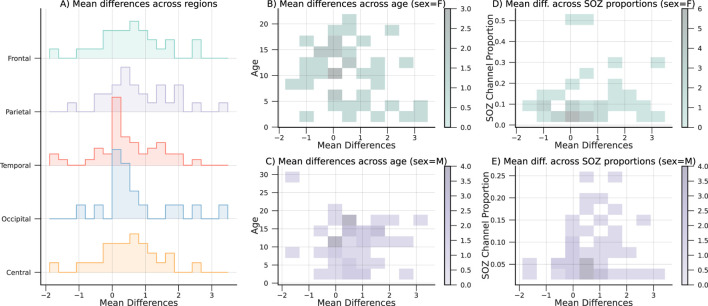
Mean CLCD differences, with respect to the **(A)** SOZ location or region, **(B,C)** age and sex, and **(C,D)** proportion of SOZ channels and sex. Note that no remarkable effect is observed between the covariates and the mean difference.

On average, most subjects in the CHM dataset have lower CLCD scores in the SOZ channels than in the nSOZ electrodes. However, 20 patients in CHM, and 2 patients in HUP, exhibit the opposite phenomenon from a total of 89 and 15 patients, respectively. We examined the possible confounding factors contributing to these disparities by analyzing the patient demographics in the CHM dataset. The results of this comparison are highlighted in Figure 10. No discernible pattern was identified in relation to age, SOZ location, or sex. Although earlier research has not explicitly examined possible differences in synchronization variability, discrepancies in the connectivity values have been reported in the literature on interictal connectivity in iEEG: Lagarde et al. found that functional connectivity is lower inside the SOZ (and the propagation zone) in comparison with the other channels (Lagarde et al., 2018). Similarly, Conrad et al. reported that lower average connectivity metrics increase “the likelihood of an electrode being in the SOZ” (Conrad et al., 2022). In contrast, Shah et al. reported increased functional connectivity in the SOZ, also associated with positive surgical outcomes (Shah et al., 2019). Higher levels of synchronization or connectivity in the SOZ channels have also been found in patients with focal cortical dysplasia by Varetto et al. (Varotto et al., 2012). The large number of subjects in our retrospective study may have emphasized this contrasting difference. Nevertheless, our analysis recognized the classification potential through the separation metrics, despite this effect. Thus, this reemphasizes the hypothesis that SOZ channels have a unique connectivity structure that differentiates them from the other channels.

This study aims to analyze the potential use of our method in facilitating the identification of SOZ channels. However, we can also evaluate the relationship between our metric and the surgical outcome after an intervention using the details provided in the HUP dataset. Our current findings reveal that favorable outcomes (Engel I) are associated with larger CLCD values (Figure 7D). Furthermore, assuming that the SOZ channels have been externally identified with other clinical procedures, our CLCD score can also provide an enhanced prediction. Thus, CLCD absolute differences, Cohen’s d, and separability (ROC-AUC) are statistically significantly higher (rank-sum test, *p*-value ≤ 1e-5 in all cases) in patients that had a favorable outcome in comparison with the subjects with a poor outcome (Figures 7.A–C). We may infer that the observed limitations of our metric in accurately identifying the SOZ may be inherently linked to the patient’s post-surgical recovery ability. Therefore, there is an association between the likelihood of a favorable outcome and the agreement level between our CLCD scores and the clinically determined SOZ. Additional research is required to confirm this association, as currently, there is a lack of a standard for determining the SOZ, and therefore, it is highly clinician-dependent. Further validation using resected information in a larger dataset would be critical to minimize potential biases.

## 6 Conclusion

In this paper, we propose a candidate biomarker of the SOZ that uses connectivity across binarized bursts at different frequencies to estimate a dispersion score (CLCD) for each channel. The CLCD metric quantifies the degree of similarity in the connectivity patterns that each electrode exhibits.

To minimize the effect of spikes, DC shifts, and other fast transient artifacts, we also develop a morphological burst matching (MBM) filter that uses a non-linear process to incorporate the information of two Gabor-based filters in parallel. We evaluated the performance of the MBM filter by measuring the pulse reconstruction accuracy as a function of the signal-to-noise ratio. We compared the results with FIR, Butterworth, and S-transform filters, and we observed that our proposed filter is robust against broadband artifacts.

We assessed the ability of the ripple band CLCD score (80–200 Hz) to detect SOZ clusters by retrospectively analyzing two independent open-access datasets. Those datasets were comprised of iEEG from 89 to 15 patients, respectively, with recordings sampled at approximately 1,000 Hz. Our results showed a separability between the CLCD scores in SOZ and nSOZ. Using the second dataset, which contains surgical outcomes (Engel I-IV), we found that the interquantile CLCD scores are slighly higher in patients with a positive outcome.

We acknowledge the limitations of our current validation approach, including the restriction of requiring more than one SOZ channel, the variability of our metric given that a subset of patients denoted high CLCD values in the SOZ, and our current dependence on the clinicians’ assessment for evaluating performance. We mitigated potential issues regarding inter-rater variability by evaluating two distinct datasets from different institutions. In both environments, our method produces a similar and consistent response. On top of that, our results suggested that extreme CLCD values are associated with a greater likelihood of a favorable surgical outcome. However, a comparison to the resected tissue in a larger data set would be an important future validation step, as well as a long-term evaluation of our metric’s variability. Nevertheless, our separability metrics indicate that SOZ channels have a distinctive pattern even in the small subset of patients analyzed. Although this type of behavior has been previously reported in the literature, further analysis is recommended to understand potential confounding factors that can be involved.

Overall, our findings show that CLCD is capable of identifying patterns in SOZ channels by analyzing cross-frequency connectivity. Consequently, it exhibits potential as a complementary tool to aid in surgical planning by facilitating the delineation of the SOZ.

## Data Availability

Publicly available datasets were analyzed in this study. This data can be found here: 1) iEEG on children during slow wave sleep (OpenNeuro) https://doi.org/10.18112/OPENNEURO.DS004551. V1.0.6, https://doi.org/10.1038/s41467-023-42091-y. 2) HUP iEEG dataset (https://openneuro.org/datasets/ds004100/versions/1.1.1).
