# Supplementary material for: Connectivity of high-frequency bursts as SOZ localization biomarker
Source: Front Netw Physiol. 2024 Sep 20;4:1441998. doi: 10.3389/fnetp.2024.1441998 (PMC11449702; doi:10.3389/fnetp.2024.1441998)
Supplement: Supplementary file 1 [file DataSheet2.pdf]

Connectivity of High-Frequency Bursts as SOZ Localization Biomarker
Supplementary material

Marco Pinto-Orellana [1],**Beth Lopour** [1,*]

[1] *Biomedical Engineering Department. University of California, Irvine. Irvine, California, USA.*

Correspondence*:
Beth Lopour
beth.lopour@uci.edu

1 COMPARISON WITH ALTERNATIVE METHODS

We also compared the results of our method with two different scenarios: a) when a finite impulse response (FIR) filter is used instead of our MBM filter and b) when the root-square-mean HFO detection method (Staba et al., 2002) is applied instead of our binarization process (Figure S1). Both alternative workflows

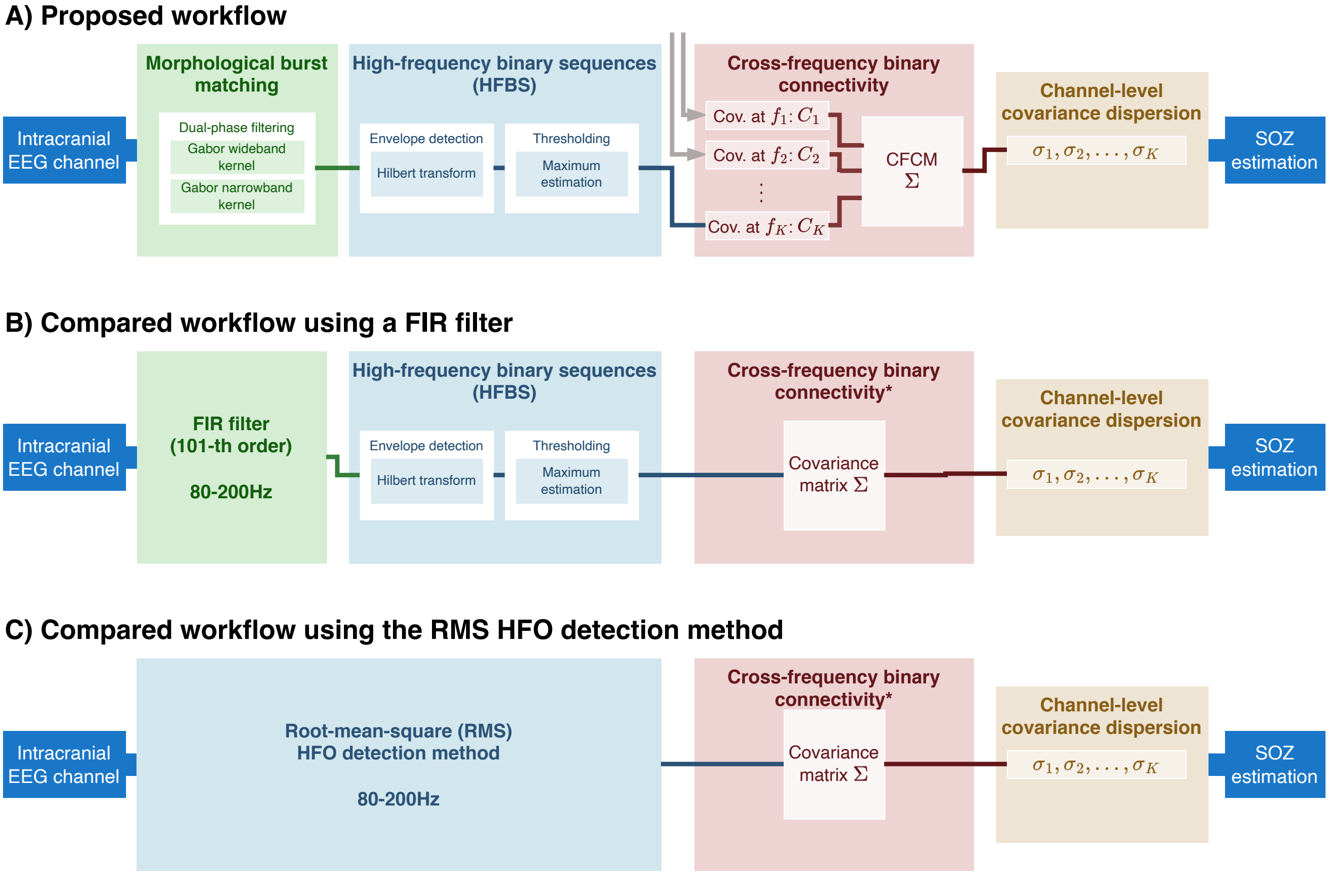

Supplementary Figure S1. Workflows compared when using A) our method, B) using an FIR filter, and C) using the RMS detection method. Note that the last two workflows do not perform analysis at specific frequencies, but over a frequency interval; however, "Cross-frequency binary connectivity" was kept in the diagrams for visualization purposes during the comparison.

Supplementary Table S1. Comparison of metrics obtained using our model, the FIR filter, and the RMS method as described in the workflows in Figure S1. ”—” denotes conditional operator.

Method	Metric	Average	Standard deviation
MBM + HFBS + CLCD	nSOZ — channels	0.0587	0.7738
	SOZ — channels	-0.5501	0.8259
	nSOZ-SOZ — subject, epoch	0.5703	0.8510
FIR(101) + HFBS + CLCD	nSOZ — channels	0.0175	0.6962
	SOZ — channels	-0.1641	0.6741
	nSOZ-SOZ — subject, epoch	0.2047	0.6040
RMS + CLCD	nSOZ — channels	-0.0397	0.7894
	SOZ — channels	0.3724	0.8411
	nSOZ-SOZ — subject, epoch	-0.4458	0.8676

were evaluated in the ripple band (80-200Hz) instead of a cross-frequency analysis (as performed in our method).

In this comparison, the standard RMS parameters resulted in unusually low detection rates of HFOs. Therefore, we adjusted the settings to a window size of 1 ms, thresholds defined as 1.5 standard deviations, minimum HFO duration of 1 ms, and a minimum number of two HFO peaks as inclusion criteria for HFO pulses. In addition, we also merged pulses that were separated by less than 1 ms.

The same separability metrics (absolute difference, ROC-AUC, and Cohen’s d) applied in the main manuscript were used for both cases. The results were compiled in Figures S2 and S3 for the FIR- and RMS-based alternative workflows, respectively.

Replacing our MBM filter with an FIR filter was associated with a loss of information about synchronization, which, in turn, lowered the ability to identify the SOZ. Furthermore, there was a considerable overlap in their distributions (Figure S2.D). Similarly, replacing our MBM filter and the binarization process with an RMS detector in the ripple band (Figure S3) resulted in a reduced capability to differentiate SOZ and nSOZ channels as their distribution also overlapped. In this scenario, the average CLCD was higher in SOZ than in nSOZ channels, i.e., the opposite phenomenon we observed with our proposed method. Moreover, we observed that the absolute mean difference was lower in both scenarios, denoting a lower ability to differentiate between SOZ and nSOZ channels. For a quantitative evaluation, the average values and their standard deviations were included in Table S1.

REFERENCES

Staba, R. J., Wilson, C. L., Bragin, A., Fried, I., and Engel, J. (2002). Quantitative Analysis of High-Frequency Oscillations (80–500 Hz) Recorded in Human Epileptic Hippocampus and Entorhinal Cortex. *Journal of Neurophysiology* 88, 1743–1752. doi:10.1152/jn.2002.88.4.1743

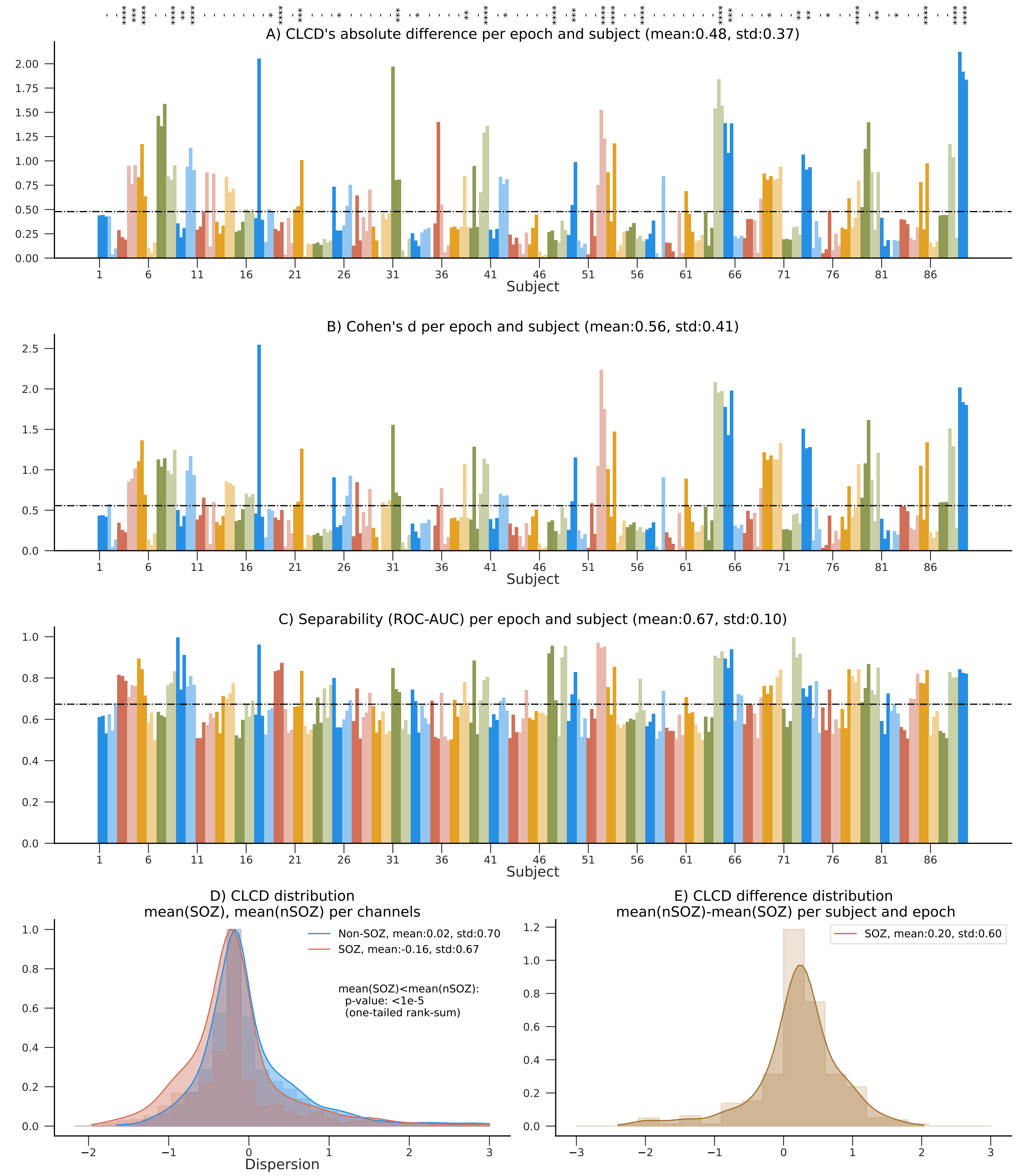

Supplementary Figure S2. Summary results on the 89 patients in the dataset using a 101-th order FIR filter in the ripple band (80-200)Hz. A) Absolute difference between average nSOZ-CLCD and SOZ-CLCD for each subject and epoch. Statistical difference is assessed using a rank-sum test (p-values: *:<0.05, **:<0.01, ***:<0.001, ****:<0.0001). B-C) Measures of separability between SOZ and nSOZ channels are shown: Cohen's d and ROC-AUC. D) Distribution of CLCD values in SOZ and nSOZ channels. The median of the nSOZ channels is statistically significantly higher than SOZ channels (rank-sum test, p-value¡1e-5). E) Distribution of the mean CLCD in nSOZ channels subtracted from the mean in SOZ channels, for all epochs and subjects.

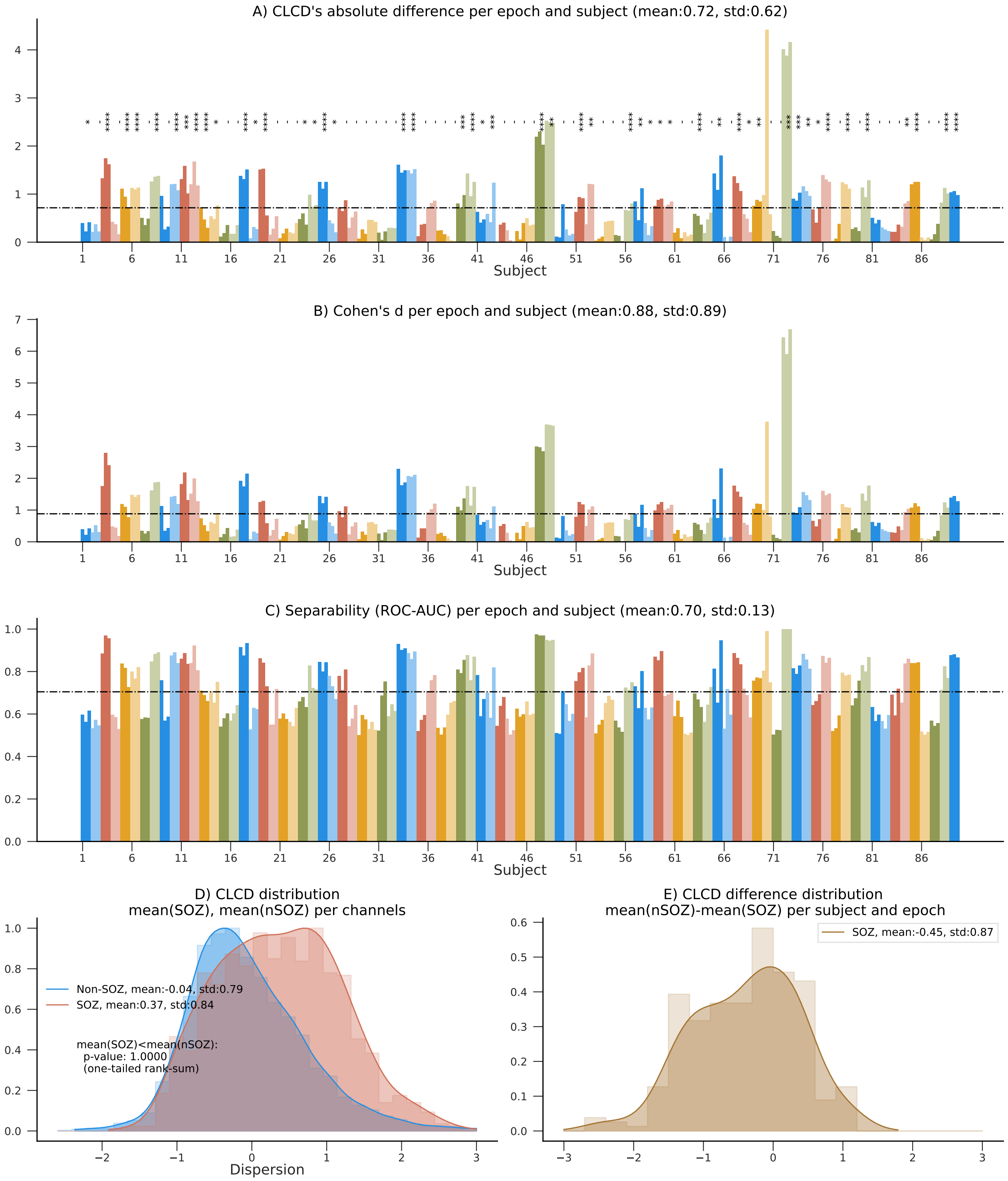

Supplementary Figure S3. Summary results on the 89 patients in the dataset using Staba's root-mean-square method in the ripple band (80-200)Hz. A) Absolute difference between average nSOZ-CLCD and SOZ-CLCD for each subject and epoch. Statistical difference is assessed using a rank-sum test (p-values: *:<0.05, **:<0.01, ***:<0.001, ****:<0.0001). B-C) Measures of separability between SOZ and nSOZ channels are shown: Cohen's d and ROC-AUC. D) Distribution of CLCD values in SOZ and nSOZ channels. The median of the nSOZ channels is not statistically significantly higher than SOZ channels (rank-sum test, p-value=1.0). E) Distribution of the mean CLCD in nSOZ channels subtracted from the mean in SOZ channels, for all epochs and subjects.
