# Supplementary material for: Connectivity of high-frequency bursts as SOZ localization biomarker
Source: Front Netw Physiol. 2024 Sep 20;4:1441998. doi: 10.3389/fnetp.2024.1441998 (PMC11449702; doi:10.3389/fnetp.2024.1441998)
Supplement: Supplementary file 2 [file DataSheet1.pdf]

Connectivity of High-Frequency Bursts as SOZ Localization Biomarker
Time-frequency properties of a Gabor kernel

Marco Pinto-Orellana [1], **Beth Lopour** [1,*]

[1] *Biomedical Engineering Department. University of California, Irvine. Irvine, California, USA.*

Correspondence*:
Beth Lopour
beth.lopour@uci.edu

This section provides an analysis of the frequency and temporal resolution of a Gabor kernel $h\left(t; f_0, \sigma\right)$, as well as its transient effects. These properties are associated with the dispersion parameter σ and can be used to configure the filter depending on the desired frequency bandwidth or the minimal number of oscillations that we anticipate the output signal should have.

1 SPECTRAL RESOLUTION

Recall that the Gabor kernel's Fourier transform (FT) is a scaled Gaussian:

$$
\begin{aligned}
H(f; f_0, \sigma) &= \frac{1}{\sqrt{2\pi}} \int_{-\infty}^{\infty} \frac{1}{\sigma\sqrt{2\pi}} e^{-\frac{1}{2}\left(\frac{t}{\sigma}\right)^2} e^{-j2\pi(f-f_0)t}\, dt \\
&= \frac{1}{2\pi\sigma} e^{-\frac{1}{2}\left(\frac{2\pi(f-f_0)}{\sigma^{-1}}\right)^2}
\end{aligned} \tag{1}
$$

where the maximum value is $(2\pi)^{-0.5}$, and it is located at f_0.

Then, we can parameterize the Gabor kernel's FT relatively to the maximum value: $H\left(f; f_0, \sigma\right) = \eta_{f_0}\sigma$ using a relative gain η_{f_0}. Additionally, a bandwidth $\Delta f = |f - f_0|$ can be defined as the interval $[f - f_0, f + f_0]$, where it is ensured that $H(f; f_0, \sigma) \geq \eta_f$. Consequently, the bandwidth can be expressed as a function of the relative gain:

$$
\Delta f = \frac{1}{2\pi\sigma}\sqrt{-2\log(\eta_f)} \tag{2}
$$

For instance, given a relative gain $\eta_{f_0} = \frac{1}{2}$, the frequency bandwidth is

$$
\Delta f \approx \frac{0.588705}{\pi\sigma} \tag{3}
$$

This expression is depicted in Table 1.

2 FILTERING BY PULSE DURATION

Assume that we have a burst signal that is a complex oscillation with the same frequency f_0 as the Gabor kernel. This input signal only exists in the time interval $[0, B\sigma]$ and is zero everywhere else. Thus, we

could formulate it as a product of the oscillatory component $e^{j2\pi f_0 t}$ and a rectangular pulse $R(t)$:

$$R(t) = \begin{cases} 1 & 0 \le t \le B\sigma \\ 0 & \text{otherwise} \end{cases} \tag{4}$$

Then, applying the Gabor kernel as a filter on the signal will produce the signal $x_B(t)$:

$$\begin{aligned} x_B(t) &= \left(R(t)e^{j2\pi f_0 t}\right) * \left(\frac{1}{\sigma\sqrt{2\pi}} e^{-\frac{1}{2}(\frac{t}{\sigma})^2} e^{j2\pi f_0 t}\right) \\ &= \frac{1}{\sigma\sqrt{2\pi}} \int_{-\infty}^{\infty} \left(e^{-\frac{1}{2}(\frac{\tau}{\sigma})^2} e^{j2\pi f_0 \tau}\right) \left(R(t-\tau)\, e^{j2\pi f_0 (t-\tau)}\right) d\tau \\ &= e^{-j2\pi f_0 t} \frac{1}{\sigma\sqrt{2\pi}} \int_{-\infty}^{\infty} e^{-\frac{1}{2}(\frac{\tau}{\sigma})^2} R(\tau - t) d\tau \\ &= e^{-j2\pi f_0 t} g_B(t) \end{aligned} \tag{5}$$

In this notation, $g_B(t)$ is an envelope described as the convolution of the rectangular pulse and the Gaussian kernel:

$$\begin{aligned} g_B(t) &= \frac{1}{\sigma\sqrt{2\pi}} \int_{-\infty}^{\infty} e^{-\frac{1}{2}(\frac{\tau - t}{\sigma})^2} R(\tau)\, d\tau \\ &= \frac{1}{\sigma\sqrt{2\pi}} \int_{0}^{B\sigma} e^{-\frac{1}{2}\left(\frac{\tau - t}{\sigma}\right)^2} d\tau = \frac{1}{\sigma\sqrt{2\pi}} \sigma \int_{-\frac{t}{\sigma}}^{B-\frac{t}{\sigma}} e^{-\frac{1}{2}v^2}\, dv = \frac{1}{\sqrt{2\pi}} \int_{\frac{t}{\sigma}-B}^{\frac{t}{\sigma}} e^{-\frac{1}{2}v^2}\, dv \\ &= \Phi\left(\frac{t}{\sigma}\right) - \Phi\left(\frac{t}{\sigma} - B\right) \end{aligned} \tag{6}$$

where $\Phi(\cdot)$ is the cumulative distribution function of a standard normal.

Then, let us evaluate the output envelope at the middle of the pulse. Due to the pulse's symmetric properties, this point is the maximum amplitude $g_{\max}$ of the output signal:

$$\begin{aligned} g_{\max} &= \Phi\left(\frac{1}{2}B\right) - \Phi\left(-\frac{1}{2}B\right) \\ &= \Phi\left(\frac{1}{2}B\right) - \left(1 - \Phi\left(\frac{1}{2}B\right)\right) \\ &= 2\Phi\left(\frac{1}{2}B\right) - 1 \end{aligned} \tag{7}$$

As a consequence, the amplitude of the burst's filtered signal $g_{\max}$ will depend solely on the duration of the pulse $B\sigma$. For instance, if the pulse has a duration 0.5σ, the output amplitude will be 0.197, while if the pulse has a duration of 5σ, the output amplitude will be 0.988.

3 TRANSIENT RESPONSE

To construct binary pulses from measured data bursts, the process outlined in Section 2 involves filtering the signal, computing the envelope, and then applying a threshold to binarize the signal. During this procedure, a transient response is introduced, which is caused by filtering the binarized signal, and it may significantly impact the calculated limits of the reconstructed pulses by introducing bias. In this section, we provide approximations of the bias effect of a Gabor filter with a specific configuration.

Consider the previous expressions associated with the rectangular pulse $R(t)$ described in the previous section. Then, evaluate the envelope at $t = -\Delta t$ (before the real start of the pulse):

$$g_B(-\Delta t) = \Phi\left(\frac{-\Delta t}{\sigma}\right) - \Phi\left(-\frac{\Delta t}{\sigma} - B\right) \tag{8}$$

To simplify this expression, we will consider two possible scenarios. First, assume that B is large enough such $\Phi(-B) \approx 0$ and $B \gg \frac{\Delta t}{\sigma}$. Then,

$$g_B(-\Delta t) \approx \Phi\left(-\frac{\Delta t}{\sigma}\right) \tag{9}$$

Note that under the assumptions of this scenario, $g_{\max} = 1$. Then, if we assume a threshold $\eta_g = \frac{g_B(-\Delta t)}{g_{\max}}$, we can determine the time prior to the pulse where the envelope has reached a relative cutoff η_g:

$$\Delta t \approx -\sigma \Phi^{-1}(\eta_g) \tag{10}$$

For instance, if we reconstruct the pulse such that it is within the interval where the signal exceeds 25% of its maximum value ($\eta_g = \frac{1}{4}$), then the estimated pulse has a bias of $\Delta t = 0.674\sigma$ on each side. Therefore, the measured burst duration after filtering will be $(B + 1.351)\,\sigma$. Conversely, if we define a considerably high threshold, $\eta_g = 0.75$, our pulse duration estimate is smaller than the ground truth, given by $(B - 1.351)\,\sigma$.

We can also consider a second scenario, when B is not sufficiently larger than $\frac{\Delta t}{\sigma}$. In this context, we can use a first-order Taylor expansion to approximate $\Phi\left(-\frac{\Delta t}{\sigma} - B\right)$:

$$\Phi\left(-\frac{\Delta t}{\sigma} - B\right) \approx \Phi\left(-\frac{\Delta t}{\sigma}\right) - B\frac{1}{\sqrt{2\pi}} e^{-\frac{1}{2}\left(\frac{\Delta t}{\sigma}\right)^2} \tag{11}$$

In a similar procedure, we can evaluate the envelope at $t = -\Delta t$:

$$\begin{aligned} g_B(-\Delta t) &\approx \Phi\left(-\frac{\Delta t}{\sigma}\right) - \Phi\left(-\frac{\Delta t}{\sigma} - B\right) \\ &= \frac{B}{\sqrt{2\pi}} e^{-\frac{1}{2}\left(\frac{\Delta t}{\sigma}\right)^2} \end{aligned} \tag{12}$$

Parameterizing the expression using the relative cutoff $\eta_g = \frac{g_B(-\Delta t)}{g_{\max}}$, we obtain a closed-form approximation for the time Δt:

$$\Delta t \approx \sigma \sqrt{-2 \log \left(\eta_g \sqrt{2\pi} \frac{\left(2\Phi\left(\frac{1}{2}B\right) - 1\right)}{B} \right)} \tag{13}$$

For instance, assuming a relative cutoff $\eta_g = \frac{1}{4}$, a burst with a duration of σ has a bias $\Delta t \approx 1.690\sigma$. Similarly, a burst with a duration of $\frac{1}{2}\sigma$ induces a bias $\Delta t \approx 1.671\sigma$.

Note that the bias in this scenario has a lower bound given by

$$\lim_{B \to 0} \Delta t = \lim_{B \to 0} \sigma \sqrt{-2 \log \left(\eta_g \sqrt{2\pi} \frac{\left(2\Phi\left(\frac{1}{2}B\right) - 1\right)}{B} \right)} = \sigma \sqrt{-2 \log(\eta_g)} \tag{14}$$

Therefore, we can unify the provided expressions based on the aforementioned assumptions:

$$\begin{cases} \Delta t \approx -\sigma \Phi^{-1}(\eta_g) & B \gg \frac{\Delta t}{\sigma} \\ \Delta t \geq \sigma \sqrt{-2 \log \eta_g} & B \ll \frac{\Delta t}{\sigma} \end{cases} \tag{15}$$

Assuming a relative cutoff of 25% ($\eta_g = \frac{1}{4}$), the bias introduced by the transient response follows the following expressions

$$\begin{cases} \Delta t \approx -\sigma \Phi^{-1}\left(\frac{1}{4}\right) = 0.674\sigma & \text{large pulses} \\ \Delta t \geq \sigma \sqrt{-2 \log \eta_g} = 1.665\sigma & \text{short pulses} \end{cases} \tag{16}$$
